# 

**Published:** 1857-08

**Authors:** 


*Veratrum Viride in Dysmenorrhœa.*—Dr. Gorham recommends in the Medical Independent, the use of Veratrum Viride. He begins two days before the expected period, and gives three drops of the tinct. every three hours, with directions to increase one drop at each succeeding dose, until nausea comes on, and then to reduce the dose again to three or four drops. In one case the nausea with retching was produced by the seven drop dose. The next case was vomited freely by the six drop dose; and the third sickened and vomited at the two drop dose. The frequency of the pulse was much reduced in all cases, and menstruation in each case came on at a later period by three or four days than the patient expected, but without the least pains, the relief appearing to be complete.

*Memphis Medical Recorder.*

